# Acute Heart Failure: Definition, Classification and Epidemiology

**DOI:** 10.1007/s11897-017-0351-y

**Published:** 2017-08-07

**Authors:** Sameer Kurmani, Iain Squire

**Affiliations:** 1 Department of Cardiovascular Sciences, University of Leicester Glenfield Hospital, Leicester, LE3 9QP UK; 2 NIHR Leicester Biomedical Research Centre, Glenfield Hospital, Leicester, LE3 9QP UK

**Keywords:** Acute heart failure, Decompensated heart failure, Challenges in acute heart failure, Definition of acute heart failure, Classification of acute heart failure, Epidemiology of acute heart failure

## Abstract

**Purpose of Review:**

The purpose of this review is to describe the extent and scope of acute heart failure (AHF), place it within its clinical context and highlight some of the difficulties in defining it as a pathophysiological entity.

**Recent Findings:**

A diagnosis of AHF is made when patients present acutely with signs and symptoms of heart failure, often with decompensation of pre-existing cardiomyopathy. The most current guidelines classify based on clinical features at initial presentation and are used to both risk stratify and guide the management of haemodynamic compromise. Despite this, AHF remains a diagnosis with a poor prognosis and there is no therapy proven to have long-term mortality benefits.

**Summary:**

We provide an introduction to AHF and discuss its definition, causes and precipitants. We also present epidemiological and demographic data to suggest that there is significant patient heterogeneity and that AHF is not a single pathology, but rather a range of pathophysiological entities. This poses a challenge when designing clinical trials and may, at least in part, explain why the results in this area have been largely disappointing.

## Introduction

Heart failure (HF) is a clinical syndrome characterised by a constellation of symptoms (dyspnoea, orthopnoea, lower limb swelling) and signs (elevated jugular venous pressure, pulmonary congestion) often caused by a structural and/or functional cardiac abnormality resulting in reduced cardiac output and/or elevated intracardiac pressures [1••]. As a disease entity, it has wide-reaching implications not only in terms of mortality and morbidity for affected individuals but also for the infrastructure required to provide care for these patients. Within the UK, an estimated £980 million is spent per year on managing HF [2] and the World Bank estimates the global economic cost at $108 billion per annum [3]. Although estimates vary depending on the study population, the prevalence of HF is approximately 1–2% and rises to >10% among people over the age of 70 years [4]. This figure may underestimate the true scale of disease as the estimated prevalence of those with asymptomatic left ventricular (LV) systolic dysfunction in those aged over 65 years is 5.5% [5]. One study estimates that the overall lifetime risk of developing HF is 33% for men and 28% for women [6].

Consensus guidelines [1••] tend to use the term HF to refer to those patients with established chronic heart failure (CHF) whose symptoms may be graded according to the New York Heart Association (NYHA) functional classification. Over the past 20–30 years, the understanding of this condition has improved significantly both from the pathophysiological perspective and from the provision of disease-modifying therapies informed by clinical studies. In contrast, the presentation and management of patients presenting with acute heart failure (AHF) is less well understood. These patients present with a rapid onset of disease, often in the context of pre-existing cardiomyopathy, and their admission to hospital heralds a poor prognosis with a high risk of readmission and death post-discharge. Data from the UK National Heart Failure Audit demonstrate mortality rates during the index admission of around 10% with a post-discharge 30-day and 1-year mortality of 6.5% and 30%, respectively [7].

In this article, we seek to provide an introduction to AHF and place it in its appropriate clinical context. Using epidemiological and demographic data, we highlight some of the challenges faced in the identification and management of this condition and the role that clinical trials have to play.

## De Novo Acute Heart Failure

Acute heart failure is broadly defined as a rapid onset of new or worsening signs and symptoms of HF [8]. It is often a potentially life-threatening condition, requiring hospitalisation, and emergency treatment is aimed predominantly at managing fluid overload and haemodynamic compromise. This umbrella term includes patients presenting for the first time with typical symptoms and signs of heart failure (de novo AHF) and also those with worsening of their pre-existing cardiomyopathy (acute decompensated heart failure).

De novo AHF occurs when there is a sudden increase in intracardiac filling pressures and/or acute myocardial dysfunction which can lead to decreased peripheral perfusion and pulmonary oedema. The most common aetiology is cardiac ischaemia where (sub)-total coronary occlusion leads to decreased contractility in myocardium subtended by the affected coronary artery. In this case, management is focussed not only on haemodynamic compromise but also on reperfusion with the aim of restoring myocardial contractile function.

A less common precipitant to AHF is one of a number of possible non-ischaemic myocardial insults. This includes the onset of acute myocardial dysfunction with inflammatory insults (e.g. viral cardiomyopathy), with toxic insults (e.g drug-induced cardiomyopathy) and indeed with insults of an undefined nature such as with peripartum cardiomyopathy. Admission to hospital with AHF can often herald a diagnosis of CHF as these acute insults may have long-term sequelae on myocardial function. Equally, patients may present with AHF in the context of reversible myocardial dysfunction such as tachycardia-induced arrhythmogenic cardiomyopathy, sympathoexcitation-induced Takotsubo cardiomyopathy and those related to endocrine disease such as the hypermetabolic state in thyroid storm. Management in these causes is aimed at not only at mitigating haemodynamic compromise during the index admission but also at the identification and correction of the underling insult.

In addition to myocardial dysfunction, AHF can be precipitated by acute valvular incompetence. This most commonly occurs in an ischaemic context (damage to the sub-valvular apparatus) leading to acute mitral regurgitation but can also occur without ischaemia per se as is the case with infective and non-bacterial thrombotic endocarditis. Extra-cardiac pathologies may also precipitate AHF as is the case with pulmonary embolism or pericardial effusion causing tamponade, both of which reduce LV output and therefore reduce peripheral perfusion

De novo AHF can therefore be precipitated by a number of causes, not exclusively with pump failure, which characteristically present with reduced perfusion pressures and pulmonary oedema. Management is aimed at supporting, either pharmacologically or mechanically, haemodynamic compromise and at correction of the underlying cause.

## Acute Decompensated Heart Failure

The large majority of patients presenting with AHF do so in the context of pre-existing cardiomyopathy, a situation described as acute decompensated heart failure (ADHF). There are a number of key differences between this group of patients and those presenting with de novo AHF that have implications for how haemodynamic compromise is assessed and how the condition is managed.

Unlike with de novo AHF, patients with ADHF tend to present with signs and symptoms of congestion and fluid retention (weight gain, exertional dyspnoea, orthopnoea, dependent oedema) rather than with pulmonary oedema or cardiogenic shock that characterise acute LV systolic dysfunction. This is the result of the chronic, often dysregulated, neuro-humoral compensatory mechanisms which act to maintain a haemodynamic status quo despite worsening LV function. Decompensation occurs when the balance tips towards fluid overload as the compensatory mechanisms prove inadequate or indeed fail all together. This is borne out by data from the IMPACT-HF registry which shows that acute decompensated heart disease takes a more insidious course and patients present to hospital in extremis following reported symptoms of congestion predating their admission by days or even weeks [9].

As with de novo AHF, decompensation of CHF can occur in a range of clinical settings. Demographic studies of patients with decompensated CHF have shown that there is a high prevalence of co-morbidities including atrial fibrillation/flutter (30–46%), valvular heart disease (44%) and dilated cardiomyopathy (25%) [10]. These conditions are in precarious balance in patients with pre-existing myocardial dysfunction, and any perturbation, for example an episode of atrial fibrillation with fast ventricular response, can trigger decompensation and admission with AHF. In fact, a number of descriptive studies including OPTIMIZE-HF have identified uncontrolled hypertension, new or worsening ischaemia, and arrhythmias (mostly atrial) as the most common co-morbidities precipitating admission to hospital in patients with pre-existent cardiomyopathy [11].

Due to the chronic nature of their underlying disease, patients presenting ADHF will invariably exhibit a number of medical co-morbidities that contribute to the onset and severity of hospital admission. Renal dysfunction and diabetes mellitus are two examples of non-cardiac co-morbidities that are highly prevalent in patients with decompensated heart disease which may adversely affect outcomes. This is borne out by observational studies of patients with ADHF in which 20–30% have an element of renal dysfunction and 40% have diabetes mellitus [10]. Furthermore, as a result of multiple co-morbidities, this patient group will invariable also exhibit polypharmacy and side effects of medications, such as non-steroidal anti-inflammatory analgesics and thiazolidinediones, may also precipitate admission. Conversely, non-compliance or cessation of medication, particularly with diuretics or those with proven prognostic benefit in CHF, has also been identified as a significant factor precipitating admission. Finally, the presence of multiple co-morbidities renders these patients much more susceptible to intercurrent infective illness such as cellulitis or exacerbations of chronic lung disease. There is a haemodynamic strain placed upon the body to fend of these illnesses, and in the context of limited cardiac reserve, this strain can be a precipitant to admission with ADHF. These factors are often complex and interlinked, and it is important to note that in 40–50% of patients admitted with ADHF, a clear underlying precipitant cannot be identified [12].

Patients with ADHF present with a more insidious onset complicated by multiple medical co-morbidities and often with congestion as their predominant clinical feature. Management is aimed at treating intercurrent precipitants and encouraging adherence to disease-modifying therapy.

## Epidemiology of Acute Heart Failure

Our knowledge of the epidemiology of AHF is informed by a number of large-scale registries conducted in the USA, including ADHERE [13••] and OPTIMIZE-HF [14], Europe including the European Heart Failure Surveys (EHFS) I [15] and II [16] and the ESC-HF Pilot Registry [17], as well as the international ALARM-HF [18] registry. This allows for a comprehensive description of patients presenting with AHF; however, there are three key demographic details to highlight. Firstly, patients are predominantly male with a mean age at presentation of >70 years which is consistent with the epidemiology of both ischaemic heart disease and CHF. Secondly, and related to the first point, the majority of patients (66–75%) have a previous history of HF and present with decompensation of their existing disease rather than de novo AHF. Finally, as discussed earlier, these patients exhibited a high burden of co-morbid disease including diabetes mellitus (up to 40% in some registries) and chronic obstructive pulmonary disease (approximately 20% of patients) [7].

In most of the published registries of AHF including ADHERE [19], OPTIMIZE [20] and the EHFS [10], the in-hospital mortality ranges from 4 to 7% with a median length of stay between 4 and 11 days. Data from the ALARM-HF registry however suggests that in-hospital mortality is approximately 11%, with a similar median length of stay which may be attributable to the higher number of patients admitted with cardiogenic shock in this study. The most recent data from the National Heart Failure Audit (2016) in the UK (7) gives a clearer picture. From 56,915 admission captured by the audit from April 2014 to March 2015, the overall in-patient mortality rate was 9.6%. However, there were significant differences in mortality rates when comparing patients aged <75 years (4.8%) to those aged >75 years (12%). Furthermore, in-hospital mortality was consistently better for patients managed in a specialist cardiology setting (7.1%) when compared to those admitted to a general medical ward (9.6%).

Mortality post-discharge continues to be high and appears not to have improved significantly over the past decade. Previous studies have demonstrated that at 1 year, mortality for patients hospitalised for AHF was in the order of 20% [21]. The ADHERE registry demonstrated a 1-year mortality as high as 36%, [13••] but once again, this may be attributable to the high proportion of patients within that registry admitted with cardiogenic shock. The National Heart Failure Audit has demonstrated an overall 1-year mortality of 29.6% in patients hospitalised for HF in the UK, and as with in-hospital mortality, this was influenced by the setting in which the patients were managed during their index admission (cardiology vs general medical) and also by the follow-up received. Those patients who were prescribed a full complement of disease-modifying therapy (ACE inhibitor/angiotensin receptor blocker, beta blocker and mineralocorticoid receptor antagonist) and those followed up in a HF disease management programme had better mortality rates at 1 year than their counterparts without these interventions [7]. These observations strongly support the provision of specialist care and judicious use of evidence-based pharmacological therapy in the long-term management of patients with AHF.

It is interesting to note that 40–55% of patients admitted with HF in the registries and 30% in the National Heart Failure Audit had normal or near-normal LV systolic function. Those patients presenting with AHF in the context of preserved LV systolic function tend to be older and female and are more likely to have hypertension as a co-morbidity [22]. HF with preserved ejection fraction (HFpEF) is consistently a significant feature in the clinical trials of AHF. In RELAX-AHF where investigators examined the effect of serelaxin in a placebo-controlled trial, only 55% of patients recruited had an ejection fraction (EF) of less than 40%. When compared to patients with HF with reduced ejection fraction (HFrEF), all-cause hospitalisations and deaths in patients with HFpEF are more likely due to non-cardiovascular disease [23] and are reflective of the demographics of this population and their extensive co-morbidities. However, the difference between patients with HFpEF and HFrEF, in terms of cardiovascular death and hospitalisations for worsening HF, are disproportionately modest given that no treatment has been shown to convincingly reduce mortality or morbidity in HFpEF. The EVEREST trial investigated the effects of oral tolvaptan on mortality outcomes in patients admitted with AHF. The trialists recruited patients, with a mean EF of 27.5% in the placebo arm, and demonstrated a 1-month cardiovascular mortality and/or hospitalisation for HF of 9–10% [24]. In contrast, the placebo arm of RELAX-AHF had a higher mean EF of 38.6% but a comparable 1-month cardiovascular death or readmission for HF (8–9%) [25]. The reasons for this are likely multifactorial and reflect a balance between the worse haemodynamic profile exhibited by patients with HFrEF (which may offset the benefits of disease-modifying therapies) and a worse pre-morbid condition in patients with HFpEF (which will negatively impact on cardiovascular outcomes), combined with all of the caveats of comparing trials powered for different endpoint. However, it may also reflect the fact that the definition and classification of patients with AHF do not preclude variability in LV systolic function and the difference in outcomes may in part be attributable to subtle differences in pathophysiological entities included under this common term.

## Classification of Acute Heart Failure

The definition of AHF presented here is broad and there have been many attempts to stratify this further [26]. Although characterised by a distinctive set of signs and symptoms, a major challenge in classifying AHF as a single entity is that the patient population is not uniform. Patients admitted with HF exhibit a wide spectrum of disease and range from those with severe LV systolic dysfunction and low cardiac output to those with severe hypertension and normal or near-normal LV systolic function. The majority of patients with AHF lie between these extremes and therefore also demonstrate a distribution of underlying pathology and precipitants, leading to the common endpoint of fluid overload.

Older guidelines by the European Society of Cardiology [27] classified patients into one of six groups (I–VI) on the basis of clinical and haemodynamic characteristics. The first three categories, namely those with ADHF (I), hypertensive AHF (II) and AHF with pulmonary oedema (III), account for >90% of presentations to hospital. Patients with ADHF typically present with mild–moderate symptoms whereas those patients with AHF and pulmonary oedema (III) have a clinical presentation dominated by respiratory distress and hypoxaemia and display a continuum of severity from low-output states (IVa) to outright cardiogenic shock (IVb). High-output failure (V) remains an uncommon cause of AHF and is associated with conditions such as anaemia, thyrotoxicosis and Paget’s disease. It generally presents with warm extremities and pulmonary congestion and in the case of systemic sepsis, hypotension. The classification system also defines a category for right-sided heart failure (VI) and is predominated by patients with pre-existing lung disease and cor pulmonale although acute myocardial ischaemia/infarction affecting the right ventricle is also included in this group.

This is a neat classification system and focusses the treating physician towards the management of the underlying cause of AHF. However, given patients often present with a range of co-morbidities, the reasons for decompensation may not be apparent at initial presentation or indeed, there may be multiple contributing factors. Practically speaking, therefore, it may be more prudent to stratify patients with AHF based on their initial clinical presentation. This allows the attending physician to identify those most at risk in order to direct specific interventions such as instituting ionotropic agents and/or mechanical circulatory support.

One such marker used to stratify patients is systolic blood pressure (SBP) on admission. In the majority of cases, patients with AHF present with either preserved (90–140 mmHg) or elevated (>140 mmHg) SBP; the latter of which imparts a more favourable prognosis. This may be due to the permissibility of vasodilator therapy which inevitably has a hypotensive effect or indeed may reflect the fact that a higher SBP is more often seen in the context of preserved LV function. Less than 10% of patients present with systolic hypotension (SBP < 90 mmHg) which carries with it a poor prognosis and thereby allows the stratification of these patients to higher dependency areas and more aggressive therapy [28].

**Fig. 1:**
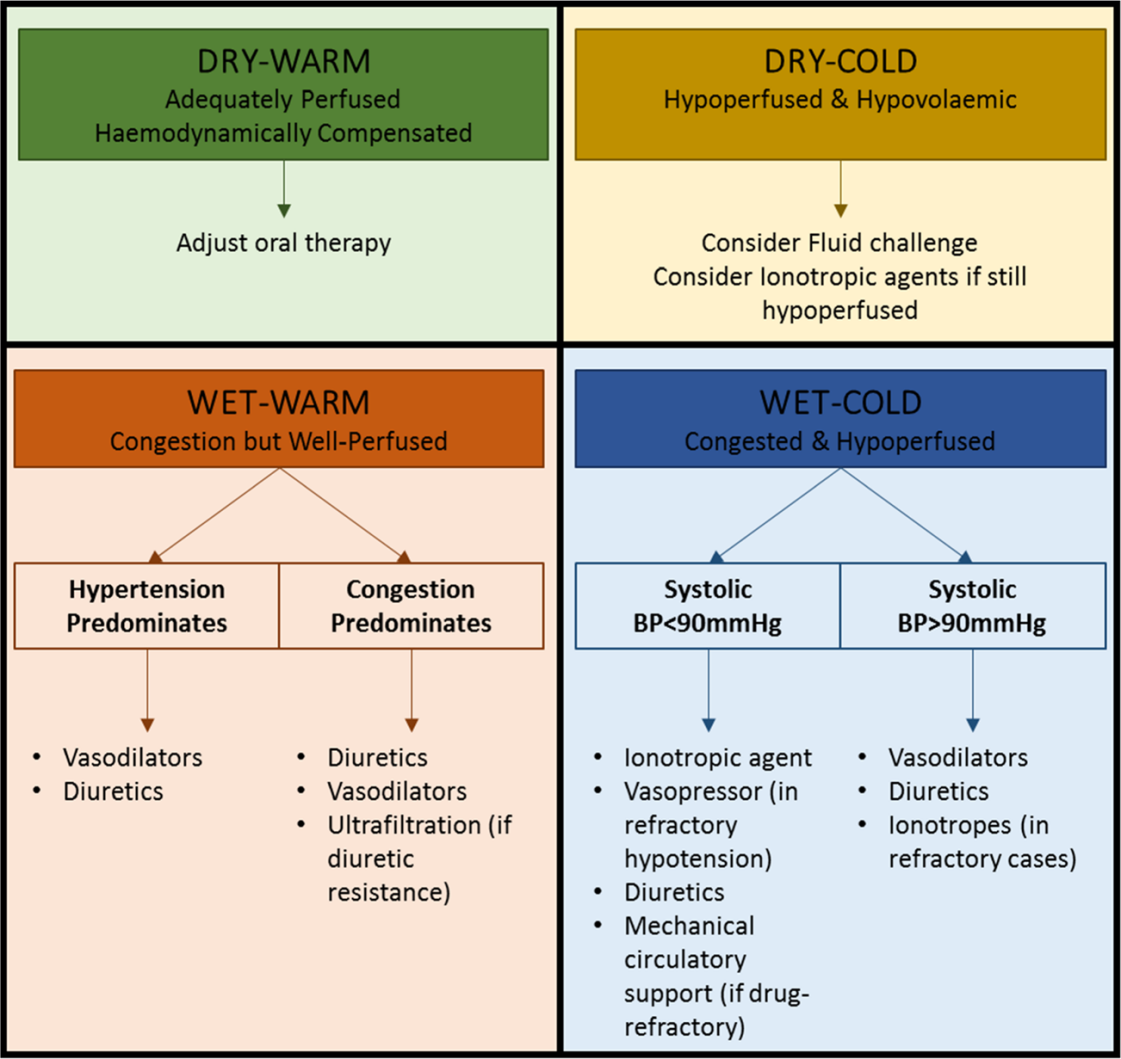
Stratification of patients admitted with AHF based on initial clinical presentation. Patients may be classified, irrespective of underlying aetiology, according to both their perfusion status (COLD vs WARM) and degree of fluid congestion (WET vs DRY). Based on the initial clinical assessment, prognosis can be determined and an appropriate management strategy put in place. It is important to note that 95% of patients presenting with AHF to the hospital have clinical features of congestion (WET). Adapted from the 2016 ESC guidelines [1••]

A more comprehensive method to classify patients presenting with AHF was developed by Stevenson and colleagues [29] and is proposed by the most recent guidelines of the European Society of Cardiology [1••]. Based on the severity of presentation rather than the underlying aetiology, this method is based on the initial clinical assessment of the patient to take into account signs and symptoms of congestion (orthopnoea, dependent oedema, elevated jugular venous pulsation) and peripheral perfusion (cold extremities, oliguria and narrow pulse pressure). Patients are described as either ‘wet’ or ‘dry’ depending on their fluid status and either ‘cold’ or ‘warm’ depending on the assessment of their perfusion status. This combined clinical assessment identifies four groups of patients (warm and wet, warm and dry, cold and dry, cold and wet) that not only allow for initial stratification as a guide to therapy (Fig. 1) but also carries with it prognostic information [1••]. Warm and dry patients have a 6-month mortality rate of 11% as compared with 40% for the cold and wet profile [29]. As a practical measure, this method of classification and risk stratification is a prudent step in the management of AHF.

## Challenges Posed by Classification and Identification

Prior to 1990, patients with stable chronic heart failure (CHF) experienced a mortality rate in the range of 60–70% [30] which has essentially halved to a current 1-year mortality of 30–40% [31]. This improvement in survival is largely attributable to the application of therapies demonstrated to provide prognostic benefit in numerous randomised controlled trials, treatments based upon an increased understanding of the pathophysiology of CHF.

However, commensurate improvements in the outcomes for patients admitted with AHF have not materialised. Data from the UK National Heart Failure Audit has revealed a 30-day mortality of approximately 15% which has remained essentially unchanged over the past 6 years [7]. Other studies have shown that the prognosis for patients hospitalised for AHF remains bleak with rates of death or recurrent hospitalisation at 6 months approaching 50% [32].

There is a notable paradox in these statistics in that despite the poor mortality outcomes, the signs and symptoms of AHF are successfully treated in the majority of patients during their index admission. This may reflect the current understanding of the pathophysiology of AHF wherein haemodynamic abnormalities result in the early features of congestion whereas end-organ damage contributes to the morbidity and mortality experienced by patients [33]. Broadly speaking, the pharmacological armament used in the treatment of AHF (loop diuretics, vasodilators and inotropes) has remained largely unchanged since the 1970s [34] and is predominantly aimed at correcting haemodynamic compromise and fluid overload. Furthermore, there has been a relative paucity of randomised placebo-controlled trials in AHF; the first of which occurred as recently as 2002 [35]. Of the studies that have been performed to date, none have presented any convincing impact of the intervention of interest on mortality rates in patients admitted with AHF.

There may be a number of different reasons for this but some centre around how the term AHF is used and by what criteria we recruit patients into trials. Randomised controlled trials in AHF usually compare treatment with a placebo and standard medical therapy. However, standard therapy has rarely been explicitly defined and, due to the nature of AHF, is often variable between patients. Large variations, often determined by clinical condition during the index admission, in the application of diuretics, inotropes, vasodilators and non-invasive ventilation may have significant effects on long-term clinical outcomes. Furthermore, the introduction, up-titration and indeed cessation of oral neurohumoral antagonists with proven prognostic benefit in CHF will inevitably vary between patients admitted with AHF, influenced by factors such as systolic blood pressure and renal function. Given the proven benefit of these drugs, it is therefore reasonable to expect that perturbations in treatment during the index admission influence long-term outcomes. Therefore, the evaluation of new therapies, especially when considering ‘hard’ clinical endpoints such as cardiovascular mortality or readmission rates, can be lost in the maelstrom of variable ‘standard’ therapy.

Another difficulty in managing patients is the variability in the diagnostic criteria used to define AHF, which thereby renders more difficult the definition of standardised inclusion criteria for studies. AHF is a clinical diagnosis based on symptoms and signs of fluid overload, with or without evidence of hypoperfusion, which may be supported by radiological evidence (pulmonary congestion on chest X-ray) and biochemical markers (B-type natriuretic peptide (BNP) or N-terminal pro-BNP). As with the assessment of symptomatology using the NYHA classification in patients with CHF, the clinical assessment of fluid overload or LV function does not necessarily correlate with the severity of symptoms. Patients may present with similar severities of pulmonary or peripheral congestion but can be classified as either ‘stable’ CHF or ‘acutely decompensated’ HF based on semi-subjective factors such as symptom severity, functional status of the patient and the infrastructure in place for providing out of hospital care. This means that when recruiting patients with AHF into trials of therapies using these clinical criteria, there is inevitably a heterogeneous population which may dilute the effect of the intervention under investigation.

A related issue is that of timing. As patients with similar clinical signs of fluid overload may present with different symptoms, patients with AHF may also present at different points in the course of their illness. To date, there is little information as to whether a therapeutic window exists in the treatment of AHF which may improve long-term outcomes and therefore, patients presenting at different stages of decompensation of cardiac function are another source of heterogeneity in clinical trials. This can be partially mitigated by the use of more objective criteria such as plasma natriuretic peptide concentrations. Data from the ADHERE registry suggests that earlier measurement of natriuretic peptides and earlier implementation of therapy may improve long-term outcomes [36]. Nevertheless, patient heterogeneity and uncertainties regarding timing of admission and intervention are ever-present when conducting these trials.

Finally, there has been a lack of consensus on appropriate endpoints for phase III studies in AHF [37]. Endpoints should be consistent, reproducible and sensitive in addition to being clinically meaningful if advancement is to be made in this field. When considering ‘hard’ outcomes such as cardiovascular mortality and readmission, there have been no trials in AHF to date that have demonstrated convincing effects in this respect in response to treatment during the index admission. More recent trials have therefore been designed to study the effects of novel interventions in AHF with respect to short-term symptoms relief, as in RELAX-HF and their use of the Likert Scale of dyspnoea [25], or shorter-term outcomes such as the use of worsening heart failure in ASCEND-HF [38]. This is most likely a recognition that there is a lack of evidence for improvement in ‘hard’ clinical endpoints and that these ‘softer’ endpoints are more achievable and clinically meaningful in the management of patients with AHF. The design of trials also belies a fundamental question related to our understanding of the pathophysiology of this condition, namely can acute intervention during the index hospitalisation improve post-discharge outcomes? This has been shown to be the case in other areas such as early reperfusion therapy in acute myocardial infarction or thrombolytic therapy in hyperacute stroke. However, to date, no short-term therapy for AHF has convincingly shown improvements in long-term mortality outcomes. Recent trials have been designed to make inroads into this very question. RELAX-AHF2 for example is a randomised, double-blind placebo-controlled phase III trial of serelaxin in patients with AHF. This drug was given in addition to standard therapy during the patient’s hospital admission, and notably, the primary endpoints are cardiovascular death and time to worsening heart failure [39•]. At the time of writing, the results of the trial are yet to be published but the study failed to show a benefit of serelaxin on the two primary endpoints. Nevertheless, the use of hard clinical endpoints in trial design is a positive step in the assessment and provision of novel disease-modifying therapy 

## Conclusions

Acute heart failure is a clinical syndrome characterised by signs and symptoms of fluid overload which require hospitalisation. Patients may present with AHF as the first presentation of heart disease but more commonly as decompensation of a pre-existing cardiomyopathy. In the latter case, admission to hospital represents a significant prognostic event in the natural history of cardiomyopathy as it is associated with worsening mortality and morbidity. The classification and subsequent treatment strategies have focussed on the management of the initial haemodynamic disturbances in a population often with multiple medical co-morbidities. However, in contrast to chronic stable heart failure, little has materialised in the way of therapies that improve long-term survival following admission with AHF. If future clinical trials are to bear fruit, their design and conduct must be done with a more comprehensive understanding of the pathophysiology and with better definition of the patient population.
